# Introduction of Inactivated Poliovirus Vaccine and Switch from Trivalent to Bivalent Oral Poliovirus Vaccine — Worldwide, 2013–2016

**Published:** 2015-07-03

**Authors:** Immunization Systems Management Group of the Global Polio Eradication Initiative

Since the 1988 World Health Assembly resolution to eradicate poliomyelitis (polio), transmission of wild poliovirus (WPV) has been interrupted in all countries except Afghanistan, Nigeria, and Pakistan (1). No polio cases caused by WPV type 2 (WPV2) have been identified since 1999, and WPV type 3 has not been detected since November 11, 2012 (2). This progress has been achieved through widespread use of oral poliovirus vaccine (OPV), most commonly trivalent OPV (tOPV), which contains types 1, 2, and 3 live, attenuated polioviruses. OPV polioviruses can undergo genetic changes during intestinal replication, and rarely, in communities with low vaccination coverage, such changes can result in vaccine-derived polioviruses (VDPVs) capable of causing paralytic polio (3). Eliminating the risk for polio caused by VDPVs will require stopping all OPV use. Among 686 cases of paralytic polio caused by circulating VDPVs (cVDPVs) that have been detected since 2006, type 2 cVDPVs (cVDPV2s) accounted for >97% (3). To eliminate the risks posed by cVDPV2s, OPV serotype 2 will be withdrawn from all immunization activities and programs through a global, synchronized replacement of all tOPV with bivalent OPV (bOPV) containing only types 1 and 3 polioviruses (4,5). This switch from tOPV to bOPV is scheduled for April 2016 (4). To reduce the risk for cVDPV2 outbreaks and to facilitate responses to outbreaks that do occur, injectable trivalent inactivated poliovirus vaccine (IPV) is being introduced into routine immunization schedules in all countries. As of June 24, 2015, 90 (46%) of 194 World Health Organization (WHO) member states were using IPV, 102 (53%) had established dates for the introduction of IPV, and two (1%) intended to introduce IPV in 2015 but had not set dates for doing so. In addition to IPV introduction in all countries, careful synchronization of the switch from tOPV to bOPV will be needed within and across all 156 countries currently using tOPV. This report summarizes progress in introducing IPV and preparations for the switch from tOPV to bOPV.

## Global Introduction of Inactivated Poliovirus Vaccine

To prepare for the global switch from tOPV to bOPV, as recommended in the Global Polio Eradication Initiative’s *Polio Eradication and Endgame Strategic Plan 2013–2018* (5), the WHO Strategic Advisory Group of Experts (SAGE) on Immunization recommended in 2012 that at least one IPV dose be introduced into routine immunization schedules in all countries (5). IPV will help protect against paralytic polio from type 2 polioviruses, provide a degree of population protection against type 2 poliovirus outbreaks, facilitate responses to any cVDPV2 outbreaks after the switch to bOPV, and aid in eradicating WPV by boosting immunity to types 1 and 3 polioviruses (5).

**FIGURE 1:**
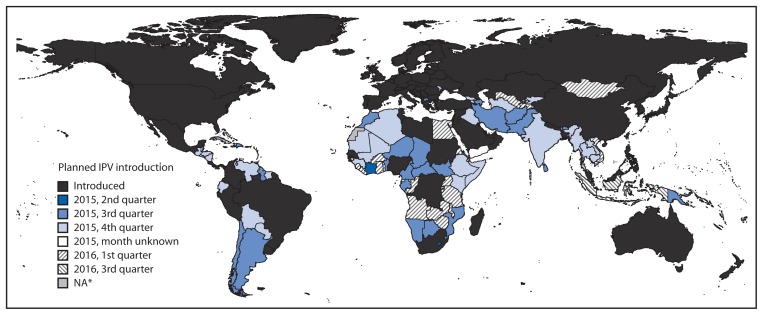
Status of introduction of inactivated poliovirus vaccine, by country — worldwide, June 24, 2015 **Source:** World Health Organization Immunization Repository. **Abbreviation:** IPV = inactivated poliovirus vaccine. * Data not available.

Among the 90 WHO member states that were using IPV as of June 24, 2015, 22 had introduced the vaccine since January 2013. In addition, 102 countries using only OPV had planned IPV introduction dates: six were planning to introduce IPV in the second quarter of 2015, 32 in the third quarter of 2015, 41 in the fourth quarter of 2015, 22 in the first quarter of 2016, and one in the third quarter of 2016 (Figure 1). Two additional countries planned to introduce IPV in 2015 but had not yet set dates for doing so.

## Global Switch from Trivalent to Bivalent Oral Poliovirus Vaccines

**FIGURE 2:**
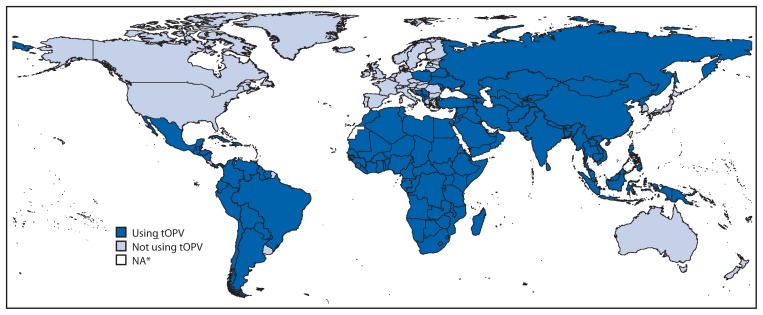
Status of trivalent oral poliovirus vaccine use, by country — worldwide, June 24, 2015 **Source:** World Health Organization Immunization Repository. **Abbreviation:** tOPV = trivalent oral poliovirus vaccine. * Data not available.

The synchronized global switch from tOPV to bOPV will affect both the routine immunization delivery systems and the supplemental immunization activities* of all 156 countries now using or stockpiling tOPV† (Figure 2). Countries using tOPV should continue to administer it until the date of the switch, with bOPV reserved only for supplemental immunization campaigns before the switch.§ Following the switch, bOPV should be exclusively used, and remaining tOPV should no longer be used and instead, should be promptly destroyed. SAGE is reviewing all preparations for the switch; in April 2015, SAGE recommended that April 2016 should be firmly planned for as the date of the switch and indicated that it would consider recommending a delay for the switch only if the risk for continued cVDPV2 transmission was deemed to be high in October 2015 (6).

During 2014, cVDPV2 circulation was detected only in Nigeria, Pakistan, and South Sudan (3). In addition, a case of cVDPV1 with onset of symptoms in September 2014 was detected in Madagascar, and in June 2015, several additional cases were linked to this outbreak through genetic testing. Persistent cVDPV2s (those circulating for >6 months) were found in both Nigeria and Pakistan, indicating ongoing weaknesses in routine immunization efforts in the affected areas. Such persistent cVDPV2s need to be eliminated before the withdrawal of tOPV. Although no cases of acute flaccid paralysis caused by cVDPV2s have been identified since December 2014, cVDPV2s have been identified from environmental samples collected in Nigeria on March 4, 2015, and in Pakistan on March 28 (3). These findings indicate that cVDPV2s were infecting persons in Nigeria and Pakistan even if they were not causing acute flaccid paralysis. Multiple supplemental immunization campaigns with tOPV are planned in all countries with an ongoing cVDPV2 outbreak or at high risk for such an outbreak (6).

WPV2 and cVDPV2 strains held in research or manufacturing facilities could also cause polio outbreaks if released into a population, and are expected to be destroyed or contained by the end of 2015, as specified in the current draft of the WHO *Global Action Plan to Minimize Poliovirus Facility-associated Risk after Type-specific Eradication of Wild Polioviruses and Sequential Cessation of Routine OPV Use* (known as GAP-III) (7). Similarly, within 3 months of the switch all type 2 Sabin poliovirus strains in manufacturing facilities using them for making the attenuated type 2 polioviruses in tOPV should be contained, and all type 2 Sabin strains in research facilities should be contained or destroyed.

To facilitate the response to any type 2 poliovirus outbreaks that occur despite these efforts, a protocol has been developed and a global stockpile of monovalent OPV type 2 is being assembled (7). Surveillance for acute flaccid paralysis cases is currently supplemented by environmental surveillance for polioviruses in sewage in at least 23 countries (8), which will help ensure that any circulation or outbreaks of type 2 poliovirus are identified and responded to quickly.

The global switch from tOPV to bOPV depends on all OPV-using countries having access to sufficient bOPV for use in routine immunization programs and in supplemental immunization activities. Although bOPV is already licensed for routine use in many countries, in others it lacks regulatory approval. Because of the April 2016 target date for the global switch to bOPV and the importance of that switch occurring in a synchronized manner, the World Health Assembly has urged countries to expedite the licensure of bOPV for use in routine immunization programs and, if the switch occurs before completion of that licensing, to temporarily allow the use of bOPV based on WHO prequalification (4).

## Discussion

The global withdrawal of tOPV, specifically its type 2 component, will represent a substantial milestone in the effort to eradicate polio, because it will mark the eradication of WPV2 and, in the long-term, should lead to the elimination of type 2 VDPVs. However, cVDPV2 outbreaks, caused either by strains that are already circulating or those that newly emerge, could occur after the switch because the number of persons susceptible to infections with type 2 polioviruses will increase over time from new birth cohorts not receiving tOPV and because multiple low income countries already have low polio vaccination coverage (9). As a result, following the switch from tOPV to bOPV, reducing the likelihood and potential extent of cVDPV2 outbreaks is essential, as is the ability to detect and respond to any such outbreaks that do occur.

Careful synchronization of the switch from tOPV to bOPV within and across OPV-using countries will be critical to minimize the risk for new cVDPV2 outbreaks. If, for example, a country continues to use tOPV after its neighbors have switched to bOPV, that country could export type 2 VDPVs to populations that are becoming increasingly susceptible to infection (9). The more tightly the switch to bOPV is synchronized, the lower the risk for new cVDPV2 outbreaks following it. Preceding the switch with high-quality tOPV supplemental immunization activities to increase population immunity in countries at risk for cVDPV2 outbreaks also will reduce the likelihood of cVDPV2 outbreaks following the switch (6,9).

The global introduction of IPV should aid in preventing paralytic polio from wild or vaccine-derived type 2 polioviruses in many persons who have received only bOPV by providing them immunity to type 2 viruses. Strengthening the routine immunization systems that distribute and administer IPV and, in case of limitations in the global IPV supply, prioritizing IPV for countries at high risk for cVDPV2 outbreaks will help maximize the impact of IPV use. Unfortunately, use of IPV alone might not always be sufficient to prevent the spread of poliovirus infections, as evidenced by the recent repeated isolation of type 1 wild polioviruses through environmental surveillance in Israel, where the population had high IPV coverage, but, because OPV had not been used since 2004, silent circulation of introduced wild polioviruses occurred (10). As tOPV is withdrawn, high quality surveillance for circulating polioviruses, both through acute flaccid paralysis surveillance and environmental surveillance, will be crucial, as will prompt, aggressive responses to any identified type 2 poliovirus outbreaks.

The global effort to introduce IPV in all countries has been facilitated by support, including technical assistance and funding for IPV purchases and operational expenses, from the Global Polio Eradication Initiative. As of June 24, 71 were receiving support provided through Gavi, the Vaccine Alliance,¶ and 18 were receiving or had been approved for support provided through WHO and the United Nations Children’s Fund (UNICEF) (7).

Through UNICEF, manufacturers are coordinating the appropriate level of production of both tOPV and bOPV, to ensure that the switch occurs as planned. The global withdrawal of the type 2 component of OPV offers a valuable opportunity to develop and test measures for conducting such a withdrawal efficiently and safely, including measures related to vaccine procurement and stock management, which also will be needed during the eventual global withdrawal of all OPV after eradication of all wild polioviruses.

**Summary**

What is already known on this topic?

No cases of poliomyelitis caused by wild poliovirus type 2 have been detected since 1999, but hundreds of cases of paralytic polio have been caused by circulating vaccine derived poliovirus type 2 since 2006. As a result, the type 2 component of oral poliovirus vaccine is slated for global withdrawal through a switch from trivalent oral poliovirus vaccine (tOPV) to bivalent oral poliovirus vaccine (bOPV).

What is added by this report?

tOPV is currently being used or stockpiled in 156 countries, all of which will need to switch from tOPV to bOPV. Inactivated poliovirus vaccine (IPV) is currently being used in the routine immunization programs of 90 countries, and because of the switch, 102 additional countries have set dates for introducing IPV. The World Health Assembly has asked that all countries currently using oral poliovirus vaccine prepare for the global switch from tOPV to bOPV in April 2016.

What are the implications for public health practice?

Because of the progress made in eradicating polio, all 156 countries using or stockpiling tOPV need to fully prepare to execute the synchronized switch from tOPV to bOPV in April 2016, one of the largest coordinated public health efforts in history, to best protect the world’s children against outbreaks of poliomyelitis caused by circulating vaccine-derived poliovirus type 2.

## Footnotes

* Supplemental immunization activities are mass vaccination campaigns conducted in a short period (days to weeks) during which a dose of OPV is administered to all children aged <5 years, regardless of previous vaccination history. Campaigns can be conducted nationally or in portions of a country.

† Israel is administering bOPV in its immunization activities but is maintaining a stockpile of tOPV.

§ bOPV is sometimes used in supplemental immunization activities focused on dealing with outbreaks of types 1 or 3 polioviruses. In general, countries should continue to administer tOPV until the switch from tOPV to bOPV to maximize population immunity to type 2 polioviruses.

¶ Formerly known as the Global Alliance for Vaccines and Immunization.

## References

[1] Hagan JE, Wassilak SG, Craig AS (2015). Progress toward polio eradication—worldwide, 2014–2015. MMWR Morb Mortal Wkly Rep.

[2] Kew OM, Cochi SL, Jafari HS (2014). Possible eradication of wild poliovirus type 3—worldwide, 2012. MMWR Morb Mortal Wkly Rep.

[3] Diop OM, Burns CC, Sutter RW, Wassilak SG, Kew OM (2015). Update on vaccine-derived polioviruses—worldwide, January 2014–March 2015. MMWR Morb Mortal Wkly Rep.

[4] World Health Organization World Health Assembly resolution: poliomyelitis.

[5] Global Polio Eradication Initiative (2013). Polio eradication and endgame strategic plan 2013–2018.

[6] World Health Organization (2015). Meeting of the Strategic Advisory Group of Experts on Immunization, April 2015: conclusions and recommendations. Wkly Epidemiol Rec.

[7] World Health Organization (2015). Type 2 OPV withdrawal: update on readiness and preparations for the switch [background paper].

[8] Porter KA, Diop OM, Burns CC, Tangermann RH, Wassilak SG (2015). Tracking progress toward polio eradication—worldwide, 2013–2014. MMWR Morb Mortal Wkly Rep.

[9] Thompson KM, Duintjer Tebbens RJ (2014). Modeling the dynamics of oral poliovirus vaccine cessation. J Infect Dis.

[10] Kopel E, Kaliner E, Grotto I (2014). Lessons from a public health emergency—importation of wild poliovirus to Israel. N Engl J Med.

